# The anti-tumor effect of the quinoline-3-carboxamide tasquinimod: blockade of recruitment of CD11b^+^ Ly6C^hi^ cells to tumor tissue reduces tumor growth

**DOI:** 10.1186/s12885-016-2481-0

**Published:** 2016-07-11

**Authors:** Adnan Deronic, Tomas Leanderson, Fredrik Ivars

**Affiliations:** Immunology group, Section for Immunology, Department of Experimental Medical Science, Lund University, Lund, Sweden

**Keywords:** Tumor growth, Small molecule, Inhibitor, Monocyte, Recruitment

## Abstract

**Background:**

Previous work has demonstrated immunomodulatory, anti-tumor, anti-metastatic and anti-angiogenic effects of the small molecule quinoline-3-carboxamide tasquinimod in pre-clinical cancer models. To better understand the anti-tumor effects of tasquinimod in transplantable tumor models, we have evaluated the impact of the compound both on recruitment of myeloid cells to tumor tissue and on tumor-induced myeloid cell expansion as these cells are known to promote tumor development.

**Methods:**

Mice bearing subcutaneous 4 T1 mammary carcinoma tumors were treated with tasquinimod in the drinking water. A BrdU-based flow cytometry assay was utilized to assess the impact of short-term tasquinimod treatment on myeloid cell recruitment to tumors. Additionally, long-term treatment was performed to study the anti-tumor effect of tasquinimod as well as its effects on splenic myeloid cells and their progenitors. Myeloid cell populations were also immune-depleted by *in vivo* antibody treatment.

**Results:**

Short-term tasquinimod treatment did not influence the proliferation of splenic Ly6C^hi^ and Ly6G^hi^ cells, but instead reduced the influx of Ly6C^hi^ cells to the tumor. Treatment with tasquinimod for various periods of time after tumor inoculation revealed that the anti-tumor effect of this compound mainly operated during the first few days of tumor growth. Similar to tasquinimod treatment, antibody-mediated depletion of Ly6C^hi^ cells within that same time frame, caused reduced tumor growth, thereby confirming a significant role for these cells in tumor development. Additionally, long-term tasquinimod treatment reduced the splenomegaly and expansion of splenic myeloid cells during a later phase of tumor development. In this phase, tasquinimod normalized the tumor-induced alterations in myeloerythroid progenitor cells in the spleen but had only limited impact on the same populations in the bone marrow.

**Conclusions:**

Our results indicate that tasquinimod treatment reduces tumor growth by operating early after tumor inoculation and that this effect is at least partially caused by reduced recruitment of Ly6C^hi^ cells to tumor tissue. Long-term treatment also reduces the number of splenic myeloid cells and myeloerythroid progenitors, but these effects did not influence established rapidly growing tumors.

**Electronic supplementary material:**

The online version of this article (doi:10.1186/s12885-016-2481-0) contains supplementary material, which is available to authorized users.

## Background

Certain myeloid cell populations are involved in the pathogenesis of both chronic inflammation and cancer [1–3]. These myeloid cells are sensitive to environmental cues and display a high degree of plasticity. Thus, cells with a similar phenotype can either promote or suppress immune responses depending on the local microenvironment [4, 5].

To survive and expand, tumors must evade the host’s immune system [6]. Interestingly, myeloid cells located within tumor tissue are highly immunosuppressive [7–9], as a result of various signaling molecules provided by tumors and stromal cells. The most prominent myeloid cell populations located in tumors are tumor-associated macrophages (TAM) and Gr1^+^ cells. The Gr1^+^ population can be subdivided into Ly6C^hi^ monocytes and Ly6G^hi^ granulocytes [10]. The Ly6C^hi^ cells can within the tumor give rise to different populations of TAM [11, 12]. Additionally, Ly6C^hi^ and Ly6G^hi^ cells present within tumor tissue are non-proliferative, have a short half-life, and thus need to be constantly replenished [11, 13–16]. This may, however, not apply to the F4/80^hi^ TAM whose numbers can also be maintained by local proliferation [12, 17, 18].

Tumors promote the expansion of myeloid cells by producing various pro-inflammatory molecules such as GM-CSF, G-CSF, IL-1β and IL-6 [8, 9, 19, 20]. Depending on the tumor model used, these cells may expand either in the bone marrow or the spleen [9]. The expansion in the spleen creates a myeloid cell reservoir and a source of Ly6C^hi^ cells that can be recruited into tissues [21, 22]. A role of this splenic myeloid cell reservoir during tumor growth was identified, as it was shown that splenectomized mice displayed reduced tumor progression, which was associated with a decrease in Ly6C^hi^ and Ly6G^hi^ cells in the tumor area [14, 23]. In this setting, an increase in granulocyte-macrophage progenitors (GMP), that were able to generate the myeloid cells recruited to the tumor, was identified within the Lin^−^ c-kit^+^ Sca1^−^ cells in the spleen [14, 23]. A similar phenomenon was also observed in other models of inflammatory disease such as atherosclerosis and colitis [24, 25].

Quinoline-3-carboxamides (Q compounds) are small molecule immunomodulators. One such Q compound, laquinimod, is currently in a phase III cinical trial for multiple sclerosis (NCT01707992). Another Q compound, tasquinimod (ABR-215050), has shown proof of concept in castration-resistant prostate cancer [26, 27]. In pre-clinical settings, tasquinimod has been shown to potently reduce the growth of several transplantable mouse and human xenograft tumors [28–34]. The reduced tumor growth was, in some of these studies, associated with anti-angiogenic effects [28, 29, 34]. Further, this compound was also shown to modulate the function of TAM and reduce immune suppression [33, 34]. Previous work has identified S100A9 as a target for the Q compounds, which prevent S100A9 interaction with its receptors Toll-like receptor 4 (TLR4) and receptor for glycation end-products (RAGE) [35]. By binding to these receptors, S100A9, a well-known alarmin, induces the transcription of various proinflammatory genes and thus promotes inflammatory responses [36–38]. S100A9 has, however, also been implicated in tumor development as it was shown to be important for the accumulation of suppressive myeloid cells and the negative regulation of the maturation of these cells to dendritic cells [39–41].

Our previous work demonstrated that the Q compound paquinimod, which is structurally similar to tasquinimod, reduced the accumulation of Ly6C^hi^ cells and SiglecF^+^ eosinophils in a model of sterile acute inflammation [42]. Further, we could show that the ameliorating effect of this compound in acute EAE, a mouse model for multiple sclerosis, operated early during the induction phase of the autoimmune response [43]. In this model, paquinimod also reduced the immunization-induced splenic myelopoiesis. In the current study, we test the hypothesis that the anti-tumor effects of tasquinimod could in part be mediated through an impact on alterations in myelopoiesis and myeloid cell recruitment to tumors, as these cells are known to promote tumor growth by their protumorigenic properties. Herein, we provide evidence in support of this hypothesis.

## Methods

### Mice and treatment

Wild type female BALB/c and C57Bl/6 mice were purchased from Taconic Europe (Ry, Denmark). All animal experiments were performed with the permit of the local committee on the ethics of animal experiments of Malmö and Lund (permit M12-13). To study the effects of the Q compound tasquinimod, female mice at the age of 7–10 weeks were treated with tasquinimod dissolved in drinking water corresponding to a daily dose of about 25 mg/kg body weight/day. Tasquinimod was provided by Active Biotech, Lund, Sweden.

### Tumor cell lines

The 4 T1 mammary carcinoma and the B16-F10 melanoma cell lines were initially obtained from ATCC and provided to us by Active Biotech. The EG7 cell line (OVA-transfected EL4 lymphoma cell line) [44] was obtained from Dr Clotilde Thery, Institute Curie, INSERM U932, Paris, France. The cell lines were expanded, frozen in aliquots and new aliquots regularly used for the *in vivo* experiments. The cells were cultured in RPMI medium (RPMI-1640 supplemented with 10 % fetal calf serum, 10 mM HEPES, 1 mM sodium pyruvate, 100 U/ml penicillin-streptomycin and 50 μM β-mercaptoethanol; all supplements from Invitrogen Life Technologies, Paisley, UK) at 37 °C, 5 % CO_2_. For trypsinization of 4 T1 cells, trypsin-EDTA (Sigma-Aldrich, St. Louis, MO) was briefly added to cells at approx. 80 % confluence and the cells were washed with RPMI medium.

### In vivo tumor growth

Tumor cells were harvested, washed twice in PBS (Invitrogen Life Technologies) and resuspended on ice in growth factor-reduced matrigel (BD Biosceinces, San Jose, CA) at a concentration of 10^6^ cells/ml. Mice were injected s.c. in the right flank with 10^5^ cells in 100 μl matrigel and tumors were allowed to grow for up to 15 days.

In experiments where cell recruitment was studied, tumor-bearing mice were injected i.p. with a total of three injections of 2 mg 5-bromo-2’-deoxyuridine (BrdU; Sigma-Aldrich) starting at day 5 post-inoculation. The injections were given with 14 h intervals and mice were sacrificed 14 h following the last injection. In this setting, tasquinimod treatment was started 24 h before the first BrdU injection and continued until the end of the study. Seven mice were included in each group.

In experiments where tumor growth was studied, tasquinimod treatment was started at the day of tumor cell inoculation and continued either until day 7 post-inoculation or throughout the study. In some experiments, tasquinimod treatment was started at day 3 or 7 post-inoculation and continued until the end of the study. Tumors were measured with a caliper every second day starting on day 6–7 post-inoculation, when tumors were palpable. The tumor volume was calculated using the following formula: length x width^2^ × 0.4. At the end of each experiment, tumors and spleens were carefully excised and weighed. Six to ten mice were included in each group.

### Antibody-mediated depletion

Gr1^+^ or Ly6G^+^ cells were depleted by i.p. injection of 500 μg anti-Gr1 (clone RB6-8C5) or anti-Ly6G (clone 1A8) antibody (BioXCell, West Lebanon, NH), respectively. Control mice were injected with the equal amount of an isotype control antibody (clone MPC-11) (BioXCell). In experiments where tumor growth was studied in conjunction with cell depletion, tumor cells were inoculated 24 h after antibody injection. Six to seven mice were included in each group.

### Cell preparation

The dissected spleens were mashed in 70 μm cell strainers, which were washed with Hank’s balanced salt solution (HBSS) (Invitrogen Life Technologies). Tibias were crushed in a mortar and the recovered cells washed with HBSS. Tumors were cut into small pieces with a scalpel and treated with 2 mg/ml collagenase IV (Worthington, Lakewood, NJ) and 0.1 % DNase (Sigma-Aldrich) for 40 min at 37 °C. Following the enzymatic treatment, the pieces were mashed in 70 μm cell strainers. Cells were quantified using AccuCount beads (Spherotech, Lake Forest, IL).

### Antibodies and flow cytometry

The following antibodies were purchased from Biolegend (Nordic Biosite, Täby, Sweden): B220-PerCP-Cy5.5 (RA3-6B2), c-kit-APC-Cy7 (2B8), CD3ε-PerCP-Cy5.5 (145-2C11), CD11b-Alexa700 (M1/70), CD11c-APC-Cy7 (N418), CD16/32-PE (93), CD45.2-PerCP-Cy5.5 (104), CD105-PE-Cy7 (MJ7/18), CD115-APC (AFS98), CD150-APC (TC15-12 F12.2), F4/80-PE-Cy7 (BM8), Ly6G-Brilliant Violet 421 (1A8), Sca1-PacificBlue (D7) and streptavidin-Brilliant Violet 605. The following antibodies were purchased from BD Biosciences: BrdU-FITC, CD19-PerCP-Cy5.5 (1D3), Ly6C-biotin (AL-21) and SiglecF-PE (E50-2440). Cells were stained with the above antibodies in FACS buffer (PBS supplemented with 5 % fetal calf serum and 0.05 % NaN_3_ (Sigma-Aldrich)). Fixable Viability Dye-eFluor506 purchased from eBioscience (Nordic Biosite) was used to detect dead cells. For BrdU staining, the FITC BrdU Flow Kit (BD Biosciences) was used according to the manufacturer’s protocol. Analysis of stained cells was performed using the LSRII flow cytometer (BD Biosciences).

### Statistical analyses

All statistical analyses were performed using the Mann–Whitney *U* test.

## Results

### Tasquinimod reduces the recruitment of Ly6C^hi^ cells to 4 T1 tumors

**Fig. 1:**
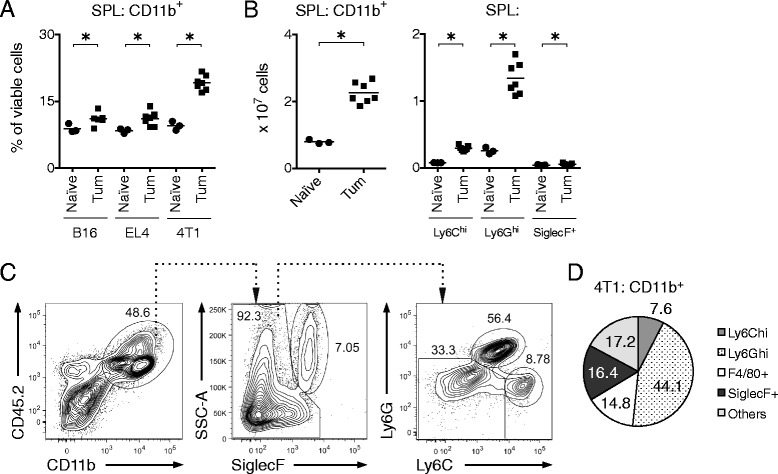
4 T1 mammary carcinoma tumors induce expansion of splenic myeloid cells. C57Bl/6 mice were inoculated with either 10^5^ B16 (*n* = 6) or 10^5^ EL4 (*n* = 7) cells and BALB/c mice were inoculated with 10^5^ 4 T1 cells (*n* = 7) s.c. Naïve C57Bl/6 or BALB/c mice (*n* = 3) were included as controls for each tumor type. Spleens and tumors were collected 7 days post-inoculation and analyzed by flow cytometry. **a**, Frequency of splenic CD11b^+^ myeloid cells gated from total viable cells. **b**, Number of splenic CD11b^+^, Ly6C^hi^, Ly6G^hi^ and SiglecF^+^ cells in naïve and 4 T1 tumor-bearing mice. **c**, Gating strategy for the identification of SSC^hi^ SiglecF^+^, Ly6C^hi^ and Ly6G^hi^ cells within the CD11b^+^ CD45.2^+^ cell population in a 4 T1 tumor. **d**, Pie chart showing the composition of the CD11b^+^ cell population in a 4 T1 tumor. Representative results of at least two independent experiments are shown for all experimental settings. *P < 0.05, Mann–Whitney *U*-test

As previous studies had indicated that Q compounds may influence recruitment of myeloid cells to sites of inflammation [42, 45, 46], we hypothesized that tasquinimod might similarly reduce the recruitment of pro-tumorigenic myeloid cells to tumor tissue. Tasquinimod was also shown to possess anti-angiogenic effects [28, 29, 34]. We expected that both recruitment of myeloid cells to the tumor *per se* and pro-angiogenic functions of these cells might be crucial during the early phase of tumor growth. Therefore, we decided to initially focus our analyses on the first 7 days of tumor development. Pro-tumorigenic myeloid cells accumulating within tumors in part originate from expanded myeloid cell reservoirs in the spleen [14, 16, 23]. To address our hypothesis, we first analyzed the accumulation of splenic myeloid cells in mice inoculated with either B16 melanoma, EL4 lymphoma or 4 T1 mammary carcinoma tumor cells (Fig. 1a), models in which tasquinimod has shown efficacy on tumor growth [31, 33, 34].

There was a statistically significant expansion of splenic myeloid cells for all three tumor models 7 days after inoculation, but the expansion was most pronounced in 4 T1 tumor-bearing mice, in which Ly6G^hi^ cells were the dominant myeloid subpopulation (Fig. 1b). In the CD11b^+^ population within the tumor tissue, similarly to the splenic CD11b^+^ population, SSC^hi^ SiglecF^+^ eosinophils, Ly6C^hi^ monocytes and Ly6G^hi^ neutrophils were identified according to the gating strategy in Fig. 1c. A population characterized as Ly6C^low^ Ly6G^low^ was also detected and approximately one third of these cells were identified as F4/80^+^ macrophages. The composition of the CD11b^+^ cell population in the 4 T1 tumor is summarized in Fig. 1d.

Previous studies showed that Ly6C^hi^ and Ly6G^hi^ cells do not proliferate within tumors and that these cells are instead replenished by recruitment from the periphery [11, 13, 14, 16]. We therefore utilized BrdU pulse-labeling as a tracker to enable the detection of recently divided cells that have been newly recruited to tumor tissue. Based on those previous studies, Ly6C^hi^ and Ly6G^hi^ cells present in tumors at the time of the BrdU pulse would not be expected to incorporate BrdU.

**Fig. 2:**
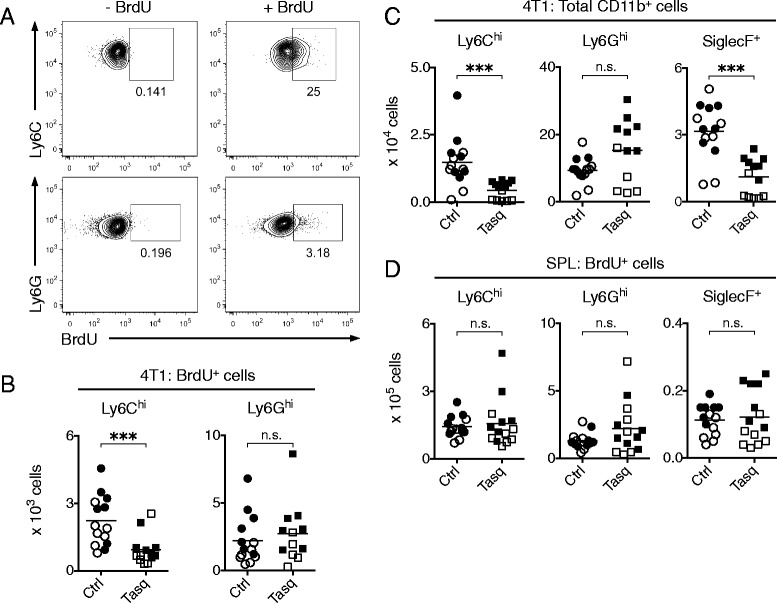
Tasquinimod reduces the recruitment of Ly6C^hi^ cells to 4 T1 tumors. BALB/c mice were inoculated with 10^5^ 4 T1 cells s.c. Tasquinimod (25 mg/kg/day) was given in the drinking water starting on day 4 post-inoculation and BrdU (2 mg) was injected i.p. starting at day 5 post-inoculation. Three injections of BrdU were given with regular intervals and the last injection 14 h before sacrifice. Spleens and tumors were collected 7 days post-inoculation and analyzed by flow cytometry. **a**, Representative FACS plots showing the efficiency of BrdU labeling of Ly6C^hi^ and Ly6G^hi^ cells in a 4 T1 tumor. **b**, Number of tumor BrdU^+^ Ly6C^hi^ and BrdU^+^ Ly6G^hi^ cells gated from CD11b^+^ CD45.2^+^ cells as in Fig. 1c. **c**, Number of total tumor Ly6C^hi^, Ly6G^hi^ and SiglecF^+^ cells. **d**, Number of splenic BrdU^+^ Ly6C^hi^, BrdU^+^ Ly6G^hi^ and BrdU^+^ SiglecF^+^ cells in 4 T1 tumor-bearing mice. Pooled data (indicated by open and filled symbols, respectively) from two independent experiments are shown (ctrl, *n* = 14; tasq, *n* = 14). n.s. = not significant, ***P < 0.001, Mann–Whitney *U*-test

We administered BrdU by i.p. injections on days 5 and 6 post-4 T1 tumor cell inoculation. To assess the impact of tasquinimod on cell recruitment, one cohort of these mice was treated with the compound on days 4 to 7 post-inoculation. Fourteen hours after the final BrdU pulse, approx. 25 % of the Ly6C^hi^ cells in the tumor were BrdU-labeled while only a minor fraction of the Ly6G^hi^ cells were labeled (Fig. 2a). Similar fractions of these cell populations were also labeled in the spleen; however, very few BrdU^+^ SiglecF^+^ and F4/80^+^ cells were detected in either compartment (Additional file 1: Figure S1A). Importantly, mice treated with tasquinimod showed a significantly reduced number of both BrdU^+^ Ly6C^hi^ cells (Fig. 2b) and of the total (BrdU^+^ and BrdU^−^) Ly6C^hi^ population (Fig. 2c). In contrast, the treatment neither affected the number of BrdU^+^ Ly6G^hi^ cells (Fig. 2b), nor the number of total Ly6G^hi^ cells within tumors (Fig. 2c).

The short-term treatment used here did not affect the absolute number of BrdU^+^ Ly6C^hi^ and SiglecF^+^ cells in the spleen (Fig. 2d), nor did it affect the total splenic populations of these cells (not shown). This excludes the possibility that the reduction of the Ly6C^hi^ and SiglecF^+^ cell populations within tumors would be due to a reduced splenic reservoir of these cells. In addition, as the reduction of these populations is selective for the cells in the tumor, it is unlikely to be caused by a toxic effect of tasquinimod operating on the cells themselves.

Taken together, these data indicate that tasquinimod specifically reduces the recruitment of Ly6C^hi^ cells into tumors. While we could also detect reduced absolute number of SiglecF^+^ cells in tumors of tasquinimod-treated mice (Fig. 2c), due to the low BrdU incorporation of these cells (Additional file 1: Figure S1A), we cannot draw firm conclusions regarding the recruitment of these cells to tumor tissue.

### Tasquinimod reduces tumor growth during the first week of tumor development

**Fig. 3:**
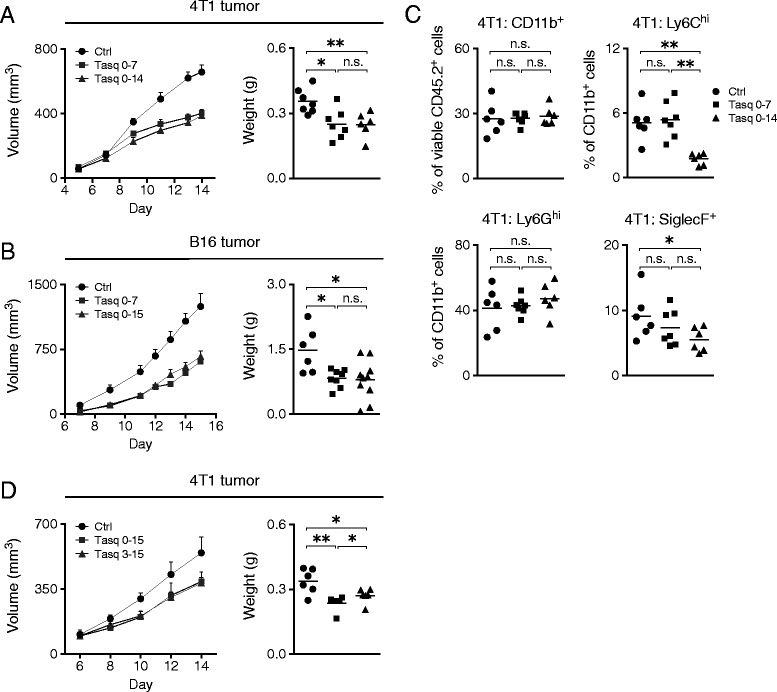
The anti-tumor effect of tasquinimod operates during the first week of tumor development. BALB/c and C57Bl/6 mice were inoculated s.c. with 10^5^ 4 T1 and 10^5^ B16 tumor cells, respectively. Tasquinimod (25 mg/kg/day) was given in the drinking water during the indicated time points. Once palpable, tumors were frequently measured throughout the study and collected at day 14–15 post-inoculation. **a**, 4 T1 tumor growth curves (*left*) and tumor weights at day 14 post-inoculation (right) (ctrl, *n* = 7; tasq 0–7, *n* = 7; tasq 0–14, *n* = 6). **b**, B16 tumor growth curves (*left*) and tumor weights at day 15 post-inoculation (*right*) (ctrl, *n* = 6; tasq 0–7, *n* = 8; tasq 0–15, *n* = 10). **c**, Frequency of CD11b^+^, Ly6C^hi^, Ly6G^hi^ and SiglecF^+^ cells gated from total viable CD45.2^+^ or CD11b^+^ CD45.2^+^ cells in 4 T1 tumors at day 14 post-inoculation. **d**, 4 T1 tumor growth curves (*left*) and tumor weights at day 15 post-inoculation (*right*) (ctrl, *n* = 6; tasq 0–15, *n* = 6; tasq 3–15, *n* = 6). Representative results of two independent experiments are shown for all experimental settings. n.s. = not significant, *P < 0.05, **P < 0.01, Mann–Whitney *U*-test

Since tasquinimod treatment reduced the number of tumor-infiltrating Ly6C^hi^ cells, that have been suggested to promote tumor growth, angiogenesis and metastasis [47, 48], we anticipated that short-term exposure to tasquinimod, in the early phase of tumor development, may impact on subsequent tumor growth. Indeed, treatment only during the first 7 days reduced tumor growth equally efficiently as treatment throughout the duration of the experiment (Fig. 3a). Also, this effect was not restricted to the 4 T1 tumor as similar results were obtained when the experiments were repeated using the B16 tumor model (Fig. 3b). These data indicate that the anti-tumor effect of tasquinimod operates during the early phase of tumor development.

The effects of tasquinimod on the composition of myeloid cells in 4 T1 tumors observed at day 7 (Additional file 1: Figure S1B), were lost in mice that were left untreated until the end of the experiment at day 14 (Fig. 3c). Mice that were treated throughout all 14 days of tumor growth, however, displayed specifically reduced frequencies of both Ly6C^hi^ and SiglecF^+^ cells within tumors (Fig. 3c). This is in contrast to the B16 tumor model where the frequency of Ly6C^hi^ cells was still significantly reduced at the end of the study, also when mice were treated with tasquinimod only during the first 7 days of tumor growth (Additional file 2: Figure S2A). These cells were thus less efficiently replenished in B16 tumors. This in turn might be a consequence of the weaker expansion of CD11b^+^ cells in the spleens of these mice (Fig. 1a). Interestingly, the reduced frequency of Ly6C^hi^ cells in 4 T1 tumors did not result in any additional anti-tumor effect, suggesting that the reduction of these cells at an early stage of tumor development is sufficient for an effect on tumor growth. As would be expected from the reduced tumor weight in tasquinimod-treated mice, the absolute number of CD11b^+^ cells was also significantly reduced (Tasq 0–7 *p* = 0.0350, Tasq 0–14 *p* = 0.0152).

To confirm that tasquinimod mainly operates on tumor growth in the early phase of tumor development, experiments with 4 T1 and B16 tumors were also performed where treatment with tasquinimod was started on day 7 post-inoculation and continued until the end of the study, at day 14. In this setting, tasquinimod failed to significantly reduce tumor growth in both tumor models (Additional file 2: Figure S2B). In the 4 T1 tumor model, there was a similar reduction in frequency of Ly6C^hi^ cells and SiglecF^+^ cells within these tumors (Additional file 2: Figure S2C), as in the experiments above (Fig. 3c). This would suggest that the effect of tasquinimod on these cell populations is not simply due to a reduced tumor burden. To verify that tasquinimod has a therapeutic anti-tumor effect, treatment of 4 T1 tumor-bearing mice was instead started on day 3 post-inoculation and continued until the end of the study, at day 15. In this setting, tasquinimod indeed displayed a significant anti-tumor effect (Fig. 3d). Thus, while tasquinimod impacts tumor growth during an early stage of tumor development, the presence of the compound at the time of tumor inoculation is not essential to induce a significant anti-tumor effect. Taken together, in the particular experimental settings used here, these results indicated that Ly6C^hi^ cells may possess protumorigenic properties in the early phase of tumor development while they do not contribute detectably in the late phase.

### Antibody-mediated depletion of Ly6C^hi^ cells reduces 4 T1 tumor growth

**Fig. 4:**
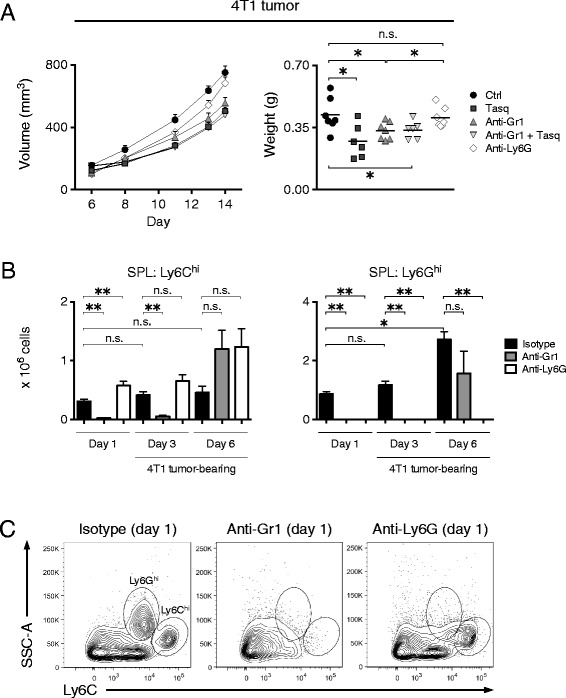
Ly6C^hi^ cells are required for 4 T1 tumor growth. BALB/c mice were inoculated with 10^5^ 4 T1 cells s.c. Gr1^+^ and Ly6G^+^ cells were depleted by the i.p. injection of 500 μg anti-Gr1 and anti-Ly6G antibody, respectively, one day prior to the inoculation of 4 T1 cells. Isotype control antibody 500 μg was injected into a control group of mice. Tasquinimod (25 mg/kg/day) was given in the drinking water from the day of inoculation and throughout the first week of tumor growth. Once palpable, tumors were frequently measured throughout the study and collected at day 14 post-inoculation. **a**, 4 T1 tumor growth curves (*left*) and tumor weights at day 14 post-inoculation (right) (ctrl, *n* = 7; tasq, *n* = 6; anti-Gr1, *n* = 7; anti-Gr1 + tasq, *n* = 6; anti-Ly6G, *n* = 7). Representative results of two independent experiments are shown. **b**, Number of splenic Ly6C^hi^ (*left*) and Ly6G^hi^ (*right*) cells 1, 3 and 6 days after cell depletion using the indicated antibodies. Pooled data from two independent experiments are shown (*n* = 4–6 for all groups). **c**, Representative FACS plots showing the efficiency of cell depletion in day 1 mice shown in **b**. n.s. = not significant, *P < 0.05, **P < 0.01, Mann–Whitney *U*-test

To verify that Ly6C^hi^ cells play an important role in the early phase of 4 T1 tumor growth, we depleted these cells using an anti-Gr1 antibody. As this depletion removes both Ly6C^hi^ and Ly6G^hi^ cells [49], another group of mice was selectively depleted of Ly6G^hi^ cells using an anti-Ly6G antibody. The antibodies were injected once, one day prior to the inoculation of mice with 4 T1 cells. Also, a group of mice were both injected with anti-Gr1 antibody and treated with tasquinimod for the first 7 days of tumor growth. As can be seen in Fig. 4a, one injection of anti-Gr1 antibody reduced tumor growth with similar efficiency as 7 days of tasquinimod treatment. Depletion using anti-Ly6G, however, had no significant impact on tumor growth, confirming the importance of the Ly6C^hi^ population in promoting tumor growth in this model. Furthermore, anti-Gr1 antibody combined with tasquinimod treatment for the first 7 days of tumor growth did not result in any additive effects (Fig. 4a). This would indicate that the effect of tasquinimod on the recruitment of Ly6C^hi^ cells to tumors is a major anti-tumor mechanism of action of this compound.

To validate the efficiency of the antibody-mediated cell depletion in this experimental setting, we analyzed mice 1, 3 or 6 days after antibody injection (Additional file 3: Figure S3A). The anti-Gr1 antibody induced a significant reduction in numbers of both Ly6C^hi^ and Ly6G^hi^ cells in the spleen already 24 h after injection (Fig. 4b). Both cell populations were still absent at day 3, but repopulated the spleen at day 6 post-depletion. Injection of anti-Ly6G antibodies also efficiently depleted the numbers of splenic Ly6G^hi^ cells within 24 h but the effect of this antibody was longer-lasting and the cells were still absent from the spleen even at day 6 post-depletion (Fig. 4b). The analyses shown in Fig. 4c, confirm that both anti-Gr1 and anti-Ly6G treatments strongly reduced the appropriate spleen cell populations, rather than causing down-modulation of epitopes that would prevent their detection in the FACS analyses. The depletion had similar effects within tumors (Additional file 3: Figure S3C) and on bone marrow cells, with the exception that repopulation was more rapid in the bone marrow (Additional file 3: Figure S3B). Thus, antibody-mediated depletion of Ly6C^hi^ cells using anti-Gr1 antibody depletes Gr1^+^ cells from the spleen for at least up to 3 days and this is sufficient for a significant anti-tumor effect.

### Long-term treatment with tasquinimod influences the tumor-induced expansion of myeloid cells in the spleen

**Fig. 5:**
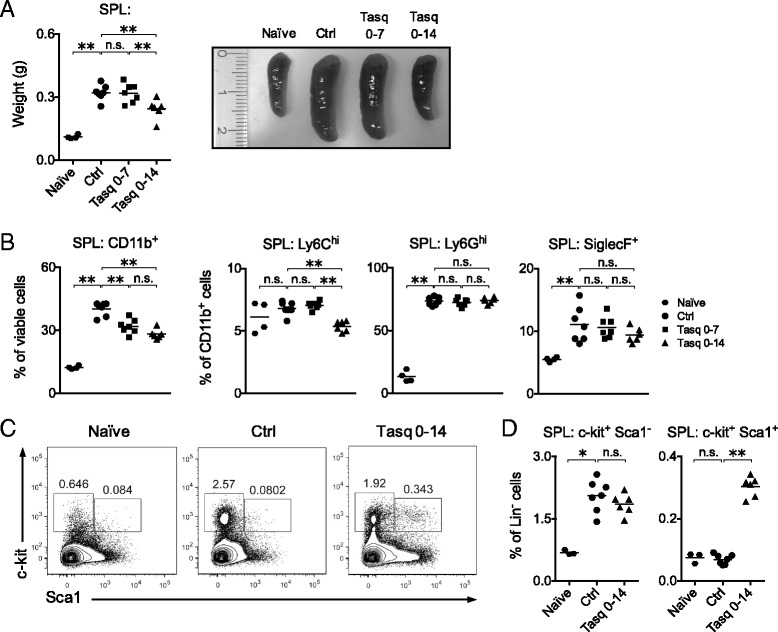
Long-term tasquinimod treatment reduces the tumor-induced expansion of splenic myeloid cells. BALB/c mice were inoculated with 10^5^ 4 T1 cells s.c. Tasquinimod (25 mg/kg/day) was given in the drinking water during the indicated time points. Spleens were collected 14 days post-inoculation, weighed and spleen cells analyzed by flow cytometry. **a**, Spleen weight (*left*) and representative image of the spleens (*right*) at day 14 post-inoculation (naïve, *n* = 4; ctrl, *n* = 7; tasq 0–7, *n* = 7; tasq 0–14, *n* = 6). **b**, Frequency of splenic CD11b^+^, Ly6C^hi^, Ly6G^hi^ and SiglecF^+^ cells gated from total viable or CD11b^+^ CD19^−^ cells. **c**, Representative FACS plots showing the gating of splenic c-kit^+^ Sca1^−^ and c-kit^+^ Sca1^+^ cells within the Lin^−^ cell population. **d**, Frequency of splenic c-kit^+^ Sca1^−^ and c-kit^+^ Sca1^+^ cells gated from Lin^−^ cells (naïve, *n* = 3; ctrl, *n* = 7; tasq 0–14, *n* = 6). Representative results of at least two independent experiments are shown for all experimental settings. n.s. = not significant, *P < 0.05, **P < 0.01, Mann–Whitney *U*-test

Since long-term treatment with the Q compounds has previously demonstrated an impact on inflammation-induced splenic myelopoiesis [43, 50], we also wanted to explore this effect further in 4 T1 tumor-bearing mice. Thus, mice were exposed to long-term treatment with tasquinimod throughout 14 days of tumor growth. These mice displayed a significant increase in spleen weight (Fig. 5a), associated with increased numbers of CD11b^+^ cells, in particular those co-expressing Ly6G (Fig. 5b). In contrast, mice treated with tasquinimod throughout the experiment displayed little splenic enlargement (Fig. 5a) and reduced frequency (Fig. 5b), as well as absolute number, of CD11b^+^ cells in the spleen (Tasq 0–14 *p* = 0.0012). Within this population, Ly6C^hi^ cells were significantly reduced and there was also a trend towards reduction of SiglecF^+^ cells. The Ly6G^hi^ cells, however, were largely unaffected (Fig. 5b). When treatment was terminated at day 7 of tumor growth, the effect on Ly6C^hi^ and SiglecF^+^ cells was lost, most likely because the spleen had been replenished with these cells following the termination of tasquinimod treatment. In summary, while short-term treatment of tasquinimod did not alter myeloid cell populations in the spleen of tumor-bearing mice (Fig. 2), long-term tasquinimod treatment influenced the splenic myeloid compartment and reduced the tumor-induced splenomegaly.

### Tasquinimod normalizes the composition of splenic myeloerythroid progenitor cells in tumor-bearing mice

Tumor-induced accumulation of myeloid cells in the spleen is at least partially a consequence of extramedullary myelopoiesis [14, 23]. Considering the effects of long-term tasquinimod treatment on splenic myeloid cells, we next evaluated the influence of tasquinimod on hematopoietic precursors in the spleen. As expected [14], there was an increased frequency of Lin^−^ c-kit^+^ Sca1^−^ cells in spleens of tumor-bearing mice at day 14 post-inoculation as compared to steady-state spleens (Fig. 5c and d). The frequency of this cell population was also increased in the spleen 6 days following depletion of Gr1^+^ cells in tumor-bearing mice, but not in isotype control-treated tumor-bearing mice (Additional file 4: Figure S4A and S4B). While tasquinimod did not alter the frequency of Lin^−^ c-kit^+^ Sca1^−^ cells, the treatment did increase the frequency of Lin^−^ c-kit^+^ Sca1^+^ cells (Fig. 5c and d). Interestingly, the frequency of cells with the same phenotype was also increased both in the spleen of anti-Gr1-treated mice (Additional file 4: Figure S4B) and in the bone marrow of tasquinimod-treated mice (Additional file 5: Figure S5B). We have not further analyzed the nature of the Lin^−^ c-kit^+^ Sca1^+^ cell population.

**Fig. 6:**
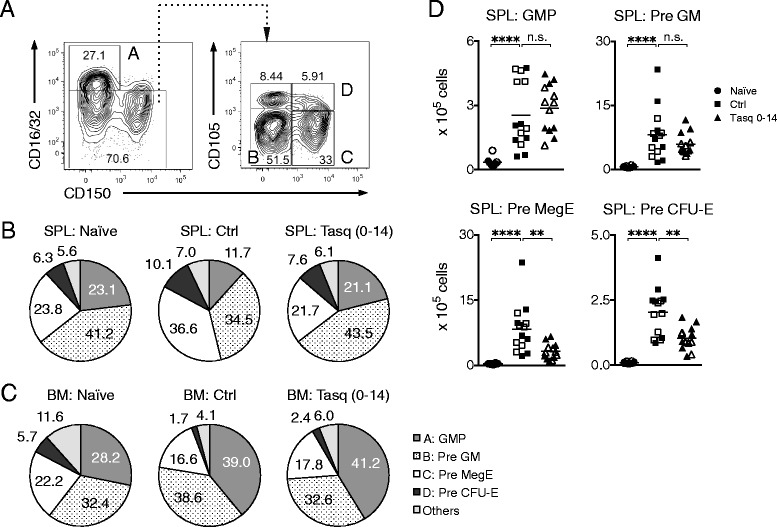
Tasquinimod normalizes the composition of splenic myeloerythroid precursor cells in tumor-bearing mice. BALB/c mice were inoculated with 10^5^ 4 T1 cells s.c. Tasquinimod (25 mg/kg/day) was given in the drinking water throughout the study. Spleens were collected 14 days post-inoculation and analyzed by flow cytometry. **a**, Gating strategy for the identification of splenic GMP (**a**), Pre GM (**b**), Pre MegE (**c**) and Pre CFU-E (**d**) cells within the Lin^−^ c-kit^+^ Sca1^−^ cell population. **b**, Pie charts showing the composition of the splenic Lin^−^ c-kit^+^ Sca1^−^ cell population. **c**, Pie charts showing the composition of the bone marrow Lin^−^ c-kit^+^ Sca1^−^ cell population. Representative results of two independent experiments are shown for **a**-**c**. **d**, Numbers of splenic myeloerythroid progenitors. Pooled data (indicated by open and filled symbols, respectively) from two independent experiments are shown (naïve, *n* = 10; ctrl, *n* = 14; tasq 0–14, *n* = 13). n.s. = not significant, **P < 0.01, ****P < 0.0001, Mann–Whitney *U*-test

Although tasquinimod did not influence the total frequency of the Lin^−^ c-kit^+^ Sca1^−^ population, we wanted to determine the potential effects of tasquinimod on distinctive myeloerythroid progenitors within this population. A study by Pronk et al. previously reported a strategy to define GMP, as well as pre-GM, pre-MegE (megakaryocyte erythrocyte progenitors) and pre-CFU-E (erythrocyte progenitors) populations within the Lin^−^ c-kit^+^ Sca1^−^ cells of the bone marrow [51]. We similarly identified these cell populations in the spleen (Fig. 6a and b). Clearly, 4 T1 tumors influenced the composition of the splenic Lin^−^ c-kit^+^ Sca1^−^ population such that the frequency of Pre MegE and Pre CFU-E cells were increased at the expense of GMP and Pre GM cells (Fig. 6b and Additional file 4: Figure S4C). This observation is in agreement with previous studies that have demonstrated the increase of megakaryocytes in the spleen of 4 T1 tumor-bearing mice as well as a relocalization of the erythropoiesis from the bone marrow to the spleen [52, 53]. Remarkably, tasquinimod completely restored the composition of myeloerythroid precursors within the Lin^−^ c-kit^+^ Sca1^−^ cells, in the spleen, to a naïve-like state (Fig. 6b and Additional file 4: Figure S4C). In terms of cell number, all analyzed myeloerythroid progenitor populations were significantly increased in tumor-bearing mice compared to naïve mice and tasquinimod again specifically reduced numbers of Pre MegE and Pre CFU-E cells (Fig. 6d).

In the bone marrow, 4 T1 tumors and tasquinimod treatment induced largely similar effects on CD11b^+^ cells as in the spleen (Additional file 5: Figure S5A). While tumor growth resulted in an increased frequency of Lin^−^ c-kit^+^ Sca1^−^ cells also in the bone marrow (Additional file 5: Figure S5B), tasquinimod displayed no impact on the total frequency of these cells and only a minor impact on the Pre GM cells within this population (Fig. 6c and Additional file 5: Figure S5C). Thus, in conclusion, tasquinimod restored the composition of myeloerythroid precursors, which accumulate in the spleen under tumor burden, to a naïve-like state. However, there was no major effect on the same populations of cells in the bone marrow, suggesting that to a certain degree, there is a compartmental specificity of tasquinimod.

## Discussion

The focus of this study was to investigate the effects of the Q compound tasquinimod on myeloid cells during tumor development. In previous reports, we have evaluated the impact of the Q compound paquinimod on myeloid cells during complete Freund’s adjuvant (CFA)-induced inflammation and could show that the expansion of these cells in the spleen was reduced [43, 50]. In particular, Ly6C^hi^ and SiglecF^+^ cells were affected [43]. In a model of necrotic cell-induced peritonitis, paquinimod also reduced the number of the same cell populations at the inflammatory site. This was not simply a result of compound toxicity, as it had no effect on these cells during steady-state conditions [42]. Previous work by others has indicated a potential effect of Q compounds on cell recruitment. One such compound, linomide, was demonstrated to impair leukocyte-endothelium interactions in a rat model of TNF-α-induced hepatic injury [45]. Another Q compound, laquinimod, was suggested to reduce the transmigration of lipopolysaccharide (LPS)-stimulated monocytes *in vitro* [46]. More recently, paquinimod was also shown to increase the rolling velocity of leukocytes on inflamed endothelium *in vivo* [54]. The exact mechanism of action of the Q compounds, and whether the target cells in our experiments are myeloid cells or endothelial cells, is still unknown. The human S100A9 protein was identified as one target molecule of paquinimod and this compound was shown to inhibit the binding of S100A9 to both of the pro-inflammatory receptors TLR4 and RAGE [35]. In a previous study, we proposed that the S100A9-TLR4 interaction may promote tumor growth [31], and as discussed therein and in more recent publications from our group [33, 34], one mode of action of tasquinimod may be to interfere with that interaction. Both the myeloid and the endothelial cells could potentially be targets for such blockade, as they both express TLR4.

Taking these findings into consideration, and the previous knowledge that Q compounds are able to reduce the growth of various tumors [28–34], we have here addressed the impact of tasquinimod on recruitment of myeloid cells to a transplantable tumor. Further, we have also investigated its impact on the accumulation of these cells in the spleen, as the spleen acts as an important reservoir of myeloid cells during tumor growth in certain tumor models [14, 16, 23]. The reason why our attention was turned to myeloid cells was due to the fact that specific myeloid cell populations, Ly6C^hi^ cells in particular, have been implicated in promoting tumor development because of their immunosuppressive and pro-tumorigenic properties [47, 48].

To analyze cell recruitment to tumors, we used an approach based on BrdU pulse labeling. It has been demonstrated not only in the 4 T1 tumor model [16], but also in other spontaneous as well as transplantable tumor models [11, 13, 14], that Ly6C^hi^ and Ly6G^hi^ cells within tumors are in a non-proliferative state and that their maintenance requires a constant input from external reservoirs [21]. For this reason, a BrdU pulse of tumor-bearing mice is likely to result in labeling of these cells only in peripheral compartments such as the spleen. Indeed, a previous study that evaluated myeloid cell proliferation in various organs of 4 T1 tumor-bearing mice, identified the spleen as the main site for this event [16]. The number of proliferating myeloid cells in the bone marrow, however, was very low and for this reason we focused our attention on BrdU^+^ cells in the spleen. We show here that short-term treatment with tasquinimod reduced the number of BrdU^+^ Ly6C^hi^ cells in the tumor, but did not affect BrdU^+^ myeloid cell populations in the spleen. These two observations, together with the previously published results [11, 13, 14], led us to conclude that tasquinimod interferes with recruitment of Ly6C^hi^ cells to the tumor rather than decreases the number of recruitable cells. Because of the differential effect of tasquinimod on Ly6C^hi^ and SiglecF^+^ cells in the spleen and tumor in these experiments, we find it unlikely that tasquinimod would have a cytotoxic effect on these cells. A caveat of this approach, however, is that not all myeloid cell populations incorporate BrdU equally efficiently. Although a major fraction of Ly6C^hi^ cells and a minor fraction of Ly6G^hi^ cells within the tumor were BrdU^+^ following the pulse, SiglecF^+^ eosinophils displayed undetectable levels of BrdU incorporation. Thus, even though the proportion of eosinophils was reduced in tumors of the treated mice, we could not formally address whether the reduction was caused by reduced recruitment of these cells.

The finding that very few F4/80^+^ cells within the tumors had detectable BrdU labeling is somewhat surprising, considering that previous reports have suggested that Ly6C^hi^ cells within tumors have the potential to differentiate to various types of TAM [11]. Furthermore, it has been suggested that differentiated TAM can proliferate [12, 17, 18]. Our data would indicate that the TAM identified in 4 T1 tumors grown for 7 days do not proliferate extensively and that very few BrdU-labeled Ly6C^hi^ cells differentiate to TAM within the time frame of the study.

To assess whether tasquinimod-mediated reduction in tumor-infiltrating Ly6C^hi^ cells could in part underlie its anti-tumor effects, we decided to temporarily deplete these cells using a specific antibody. Using this approach, we confirmed that these cells promote 4 T1 tumor growth, thereby indicating that reduction of these cells in tumors is also one anti-tumor effect of tasquinimod. Additionally, these experiments revealed that the absence of these cells in the spleen, bone marrow and tumor during the first 3 days of tumor development was sufficient to reduce tumor growth. Similarly, tasquinimod treatment for 7 days was also sufficient to reduce tumor growth. However, treatment throughout all 14 days of tumor growth did not result in an additional anti-tumor effect despite the fact that the frequency of Ly6C^hi^ cells within the tumors normalized once the treatment was terminated at day 7. Taken together, these observations indicate that the anti-tumor effect of tasquinimod is important during an early phase of tumor development.

In contrast, antibody-mediated depletion of Ly6G^hi^ cells did not significantly influence growth of the primary tumor in our experiments, suggesting that in this tumor model, these cells may be less important for initiation of tumor cell growth than Ly6C^hi^ cells. Further, long-term treatment of tumor-bearing mice with tasquinimod did not affect the frequency of splenic Ly6G^hi^ cells, but reduced the frequency of Ly6C^hi^ and SiglecF^+^ cells. Thus, tasquinimod treatment targeted the same myeloid cell populations in tumor and spleen. At present we do not know whether this observation means that the compound targets a common mechanism or independent mechanisms at these two sites. We speculate that vascular extravasation of cells in the tumor and retention of cells in spleen might both involve adhesion to endothelium and that tasquinimod could potentially interfere with certain cell-endothelial interactions at these two sites.

As previous reports have demonstrated that Ly6C^hi^ cells contribute to immune suppression, angiogenesis and metastasis in tumor-bearing mice [47, 48], it seems reasonable to assume that the reduced recruitment of Ly6C^hi^ cells during early tumor development results in an environment less hospitable for tumor growth. Indeed, tasquinimod is known to affect the tumor microenvironment in several ways [55]. Thus, tasquinimod possesses anti-angiogenic properties [28, 29, 34] and, interestingly, the tumor vasculature was significantly reduced following 1 week of treatment [34]. The anti-angiogenic effects of tasquinimod correlated with a skewing of the functional phenotype of TAM from CD206^+^ MHCII^low^ M2 macrophages to CD206^−^ MHCII^hi^ M1 macrophages [33, 34]. It is well established that M2 macrophages promote angiogenesis [56, 57]. Recent papers also demonstrated that tasquinimod treatment altered the immunosuppressive properties of CD11b^+^ cells within tumors [33, 34], which in turn may be related to the switch from M2 to M1 macrophages. We speculate that the reduced recruitment of Ly6C^hi^ cells (and potentially of eosinophils) to the tumor might disrupt the balance between M2 and M1 macrophages. Possibly, the M2 phenotype of TAM can only be maintained provided that newly arrived Ly6C^hi^ cells can continuously be polarized to this phenotype and the M1 macrophages may dominate functionally once replenishment of the M2 population is reduced. These factors might impact on the initiation of tumor cell growth or seeding, resulting in reduced growth of the tumor itself. Also, when plotting the 4 T1 and B16 tumor growth curves using a logarithmic scale, tasquinimod treatment did not influence the slope of the growth curves (not shown). This further supports that the treatment may affect the initiation of tumor cell growth or seeding rather than tumor cell proliferation *per se*. In contrast, tasquinimod treatment started at day 7, when tumors are well established, did not result in reduced tumor growth despite a reduced frequency of Ly6C^hi^ cells within the tumors. It might be argued, however, that due to the aggressive nature of 4 T1 tumors, treatment started at this stage is futile. When treatment instead was started at day 3, the anti-tumor effect of tasquinimod was maintained, indicating that there is a critical time frame between days 3–7 within which the anti-tumor effect of tasquinimod is lost.

While 4 T1 tumors in their early phase of development induced an expansion of myeloid cells in the spleen, there was no detectable splenomegaly. At a later phase, however, when tumors were more developed, the spleens were significantly enlarged and contained increased numbers of Lin^−^ c-kit^+^ Sca1^−^ hematopoietic precursor cells. This is likely the result of an increased production of myelogenic cytokines by tumor and stromal cells as well as infiltrating immune cells [8, 47]. Indeed, one previous study showed that the accumulation of Ly6G^hi^ cells in 4 T1 tumor-bearing mice is driven by tumor-produced G-CSF [20]. Recently, it was also demonstrated that G-CSF treatment could mimic the effects of the 4 T1 tumor on the spleen, as it induced splenomegaly and an increased erythropoiesis in this compartment [53]. We detected an increased frequency of cells with the Lin^−^ c-kit^+^ Sca1^+^ phenotype in spleens from both tasquinimod-treated mice and anti-Gr1-treated mice, as well as in the bone marrow of tasquinimod-treated mice. At present we do not know the nature of these cells, but based on their phenotype, they may contain hematopoietic precursors [58]. Since both these treatment regimes involve reduction of myeloid cells, we speculate that the increased frequency of these cells might be the result of some compensatory mechanism.

We further noted that the numbers of the various myeloerythroid progenitors that we analyzed in the spleen were all increased in tumor-bearing mice. In addition, the composition of the Lin^−^ c-kit^+^ Sca1^−^ population was altered in these mice, such that the frequencies of Pre MegE and Pre CFU-E cells increased at the expense of GMP and Pre GM cells. Tasquinimod treatment restored the composition of this cell population to a naïve-like state. This observation rules out the possibility that the reduction in the frequency of Ly6C^hi^ and SiglecF^+^ cells in the spleen following long-term tasquinimod treatment would be due to reduction of the number of GMP or Pre GM cells. We detected similar effects of tasquinimod on splenic myeloid cells and hematopoietic precursor cell populations when treatment of tumor-bearing mice was initiated 7 days post-inoculation, despite the lack of anti-tumor effect. Thus, the effects of tasquinimod on splenic myeloid cells and their precursors are not a result of reduced tumor burden. However, while these effects of the compound on splenic cells did not reduce the growth of already established rapidly growing tumors, it remains possible that such effects might have an impact on other, more slowly developing tumors.

In the bone marrow, tumor growth changed the composition of the Lin^−^ c-kit^+^ Sca1^−^ population such that GMP and Pre GM cells were increased at the expense of Pre MegE and Pre CFU-E cells. The absolute numbers of these cell populations, however, were not altered which correlates with previous findings that have proposed the spleen as the main site of progenitor cell expansion during 4 T1 tumor growth [16]. The effects of tasquinimod in this compartment differed from the spleen such that treatment did not completely normalize the composition of the Lin^−^ c-kit^+^ Sca-1^−^ population, but rather decreased the frequency of Pre GM cells. Further studies are required to elucidate the compartment-specific effects of tasquinimod. Recently, a subset of F4/80^hi^ VCAM-1^+^ CD169^+^ macrophages was demonstrated to support splenic myelopoiesis as well as erythropoiesis, similar to what has previously been shown for the bone marrow [53, 59, 60]. However, tasquinimod displayed no effect on number or frequency of this particular subset of macrophages in the spleen. Thus, the effects of the compound on the myeloerythroid precursors cannot be explained by an impact on numbers of these cells, but it remains possible that it may impact on their function.

## Conclusions

This study shows that tasquinimod impacts myeloid cells during tumor growth in a dual fashion. Tasquinimod reduces the recruitment of pro-tumorigenic Ly6C^hi^ cells into tumors during an early phase of tumor development without influencing the proliferation of these cells in the spleen. At a later phase of tumor development, long-term tasquinimod treatment reduces the accumulation of myeloid cells in the spleen and normalizes the composition of myeloerythroid progenitors at this site. The same progenitors in the bone marrow, however, are much less affected. Whether or not these effects are somehow connected remains to be assessed.

## Abbreviations

BrdU, 5-bromo-2’-deoxyuridine; CFA, complete Freund’s adjuvant; GMP, granulocyte macrophage progenitor; LPS, lipopolysaccharide; pre-CFU-E, erythrocyte progenitor; pre-MegE, megakaryocyte erythrocyte progenitor; RAGE, receptor for glycation end-products; TAM, tumor-associated macrophage; TLR4, Toll-like receptor 4; Q compounds, quinoline-3-carboxamides.

## Additional files

Additional file 1: Figure S1.
**A**, Representative FACS plots showing the efficiency of BrdU labeling of F4/80^+^ and SiglecF^+^ cells in a 4 T1 tumor. **B**, Frequency of the cell populations shown in Fig. 2C. (TIF 1574 kb) Additional file 2: Figure S2.
**A**, Frequency of CD11b^+^, Ly6C^hi^, Ly6G^hi^ and SiglecF^+^ cells gated from total viable CD45.2^+^ or CD11b^+^ CD45.2^+^ cells in B16 tumors at day 15 post-inoculation (ctrl, *n* = 6; tasq 0–7, *n* = 8; tasq 0–15, *n* = 9). **B**, 4 T1 tumor growth curves (left) (ctrl, *n* = 6; tasq 0–7, *n* = 7; tasq 7–14, *n* = 6) and B16 tumor growth curves (right) (ctrl, *n* = 7; tasq 0–7, *n* = 6; tasq 7–14, *n* = 7). **C**, Frequency of CD11b^+^, Ly6C^hi^, Ly6G^hi^ and SiglecF^+^ cells gated from total viable CD45.2^+^ or CD11b^+^ CD45.2^+^ cells in 4 T1 tumors at day 15 post-inoculation (ctrl, *n* = 6; tasq 0–15, *n* = 6; tasq 7–15, *n* = 7). Representative results of two independent experiments are shown for all experimental settings. n.s. = not significant, **P* < 0.05, ***P* < 0.01, Mann–Whitney *U*-test. (EPS 1130 kb) Additional file 3: Figure S3.
**A**, Experimental procedure used in Fig. 4B. **B**, Number of bone marrow Ly6C^hi^ (left) and Ly6G^hi^ (right) cells 1, 3 and 6 days after cell depletion using the indicated antibodies. **C**, Number of Ly6C^hi^ (left) and Ly6G^hi^ (right) cells in 4 T1 tumors 3 and 6 days after cell depletion using the indicated antibodies. Pooled data from two independent experiments are shown (*n* = 4-6 for all groups). n.s. = not significant, **P* < 0.05, ***P* < 0.01, Mann–Whitney *U*-test. (EPS 956 kb) Additional file 4: Figure S4.
**A**, Representative FACS plots showing the gating of splenic c-kit^+^ Sca1^−^ and c-kit^+^ Sca1^+^ cells within the Lin^−^ cell population in naïve (left), tumor-bearing, isotype-treated (middle) and tumor-bearing, Gr1^+^-depleted mice (right). **B**, Frequency of splenic c-kit^+^ Sca1^−^ and c-kit^+^ Sca1^+^ cells gated from Lin^−^ cells (*n* = 7 for all groups). Representative results of two independent experiments are shown for Additional file 4: Figure S4A and B. **C**, Frequency of splenic myeloerythroid progenitors. Individual data points from Fig. 6B are shown. Pooled data (indicated by open and filled symbols, respectively) from two independent experiments are shown (naïve, *n* = 10; ctrl, *n* = 14; tasq 0–14, *n* = 13). n.s. = not significant, **P* < 0.05, ***P* < 0.01, ****P* < 0.001, *****P* < 0.0001, Mann–Whitney *U*-test. (EPS 9412 kb) Additional file 5: Figure S5.
**A**, Frequency of bone marrow CD11b^+^, Ly6C^hi^, Ly6G^hi^ and SiglecF^+^ cells gated from total viable or CD11b^+^ CD19^−^ cells (naïve, *n* = 3; ctrl, *n* = 7; tasq 0–14, *n* = 6). **B**, Frequency of bone marrow c-kit^+^ Sca1^−^ and c-kit^+^ Sca1^+^ cells gated from Lin^−^ cells. Representative results of two independent experiments are shown for Additional file 5: Figure S5A and B. **C**, Frequency of bone marrow myeloerythroid progenitors. Individual data points from Fig. 6C are shown. Pooled data (indicated by open and filled symbols, respectively) from two independent experiments are shown (naïve, *n* = 10; ctrl, *n* = 14; tasq 0–14, *n* = 13). n.s. = not significant, **P* < 0.05, ***P* < 0.01, *****P* < 0.0001, Mann–Whitney *U*-test. (EPS 1036 kb)
