# Erratum to: The anti-tumor effect of the quinoline-3-carboxamide tasquinimod: blockade of recruitment of CD11b^+^ Ly6C^hi^ cells to tumor tissue reduces tumor growth

**DOI:** 10.1186/s12885-016-2649-7

**Published:** 2016-08-08

**Authors:** Adnan Deronic, Sahar Tahvili, Tomas Leanderson, Fredrik Ivars

**Affiliations:** Immunology group, Section for Immunology, Department of Experimental Medical Science, Lund University, Lund, Sweden

## Erratum

After publication of this work [1], it was noted that co-author Sahar Tahvili was inadvertently missed from the author list. This has been corrected here, and in the original article. The publisher would like to apologize for any inconvenience this oversight may have caused.
