# Supplementary figures and images for: The anti-tumor effect of the quinoline-3-carboxamide tasquinimod: blockade of recruitment of CD11b+ Ly6Chi cells to tumor tissue reduces tumor growth
Source: BMC Cancer. 2016 Jul 11;16:440. doi: 10.1186/s12885-016-2481-0 (PMC4939705; doi:10.1186/s12885-016-2481-0)

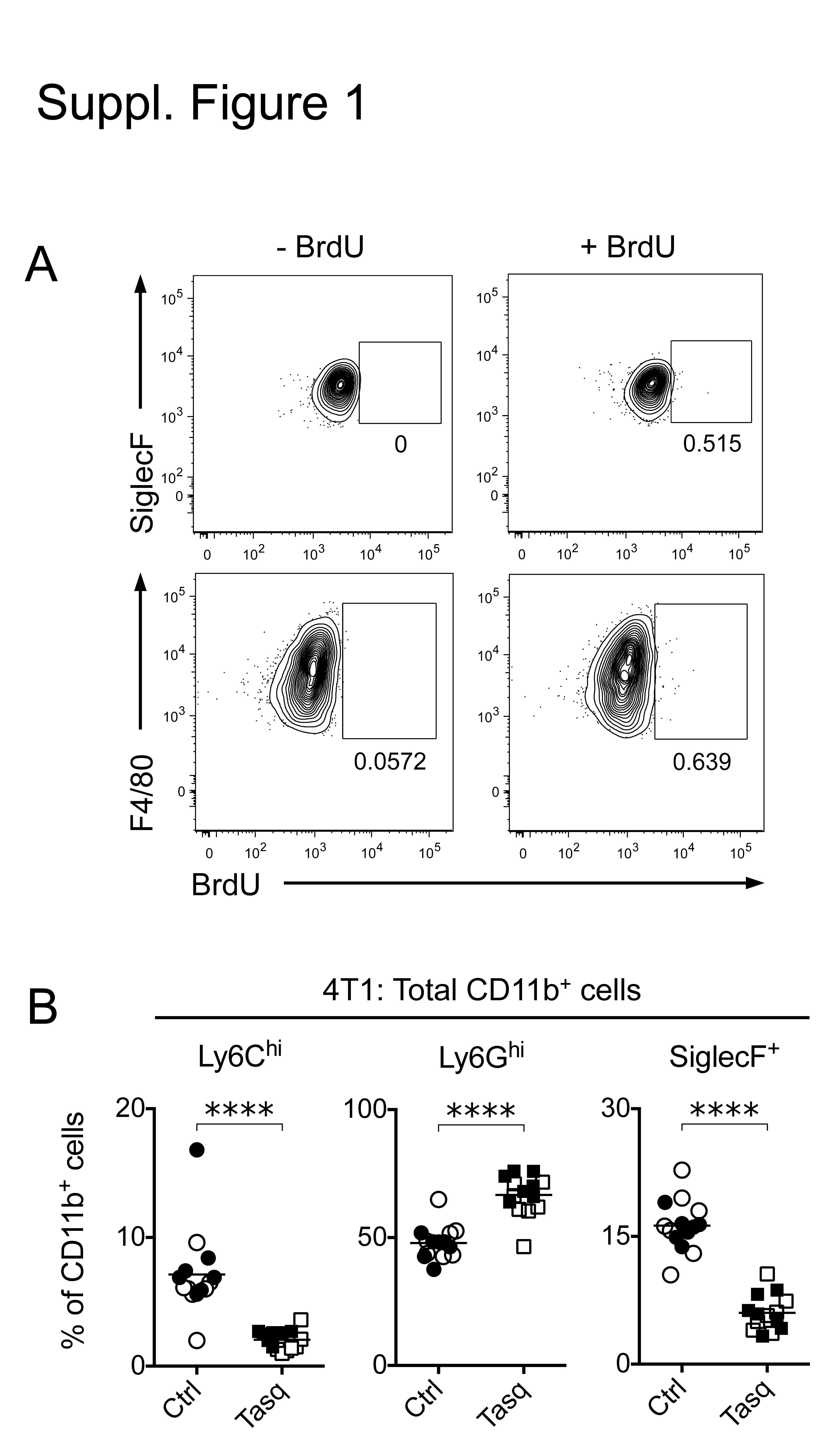

Supplement: Additional file 1: Figure S1. — A, Representative FACS plots showing the efficiency of BrdU labeling of F4/80+ and SiglecF+ cells in a 4 T1 tumor. B, Frequency of the cell populations shown in Fig. 2C. (TIF 1574 kb) [file 12885_2016_2481_MOESM1_ESM.tif]
